# Primary Malignant Melanoma of the Urinary Bladder

**DOI:** 10.7759/cureus.14067

**Published:** 2021-03-23

**Authors:** Elizabeth Snajdar, Andrina R Ajo, Katie Rosen, Roy Miller, Safi Mohammed, Craig Gordon, John C Pui, Gregory McIntosh

**Affiliations:** 1 Department of Urology, McLaren Macomb Hospital, Macomb, USA; 2 College of Natural Science, Michigan State University, East Lansing, USA; 3 Department of Hematology and Oncology, Beaumont Hospital, Farmington Hills, USA; 4 Department of Pathology, Beaumont Hospital, Farmington Hills, USA; 5 Department of Urology, Michigan Institute of Urology, Macomb, USA

**Keywords:** cystectomy, melanoma, primary malignant melanoma, extracutaneous melanoma, bladder cancer, transurethral resection of bladder tumor

## Abstract

There are only 30 reported cases of primary malignant melanoma of the bladder in the literature so far. Of those, 17 cases were reported as deceased within three years of presentation. Our case reported here is that of a 78-year-old female who presented with a new-onset incontinence and intermittent hematuria. She had no evidence of primary melanoma anywhere else in her body. The patient was treated with cystectomy and ileal conduit with plans for adjuvant chemotherapy. Unfortunately, the patient succumbed to her disease with diffuse metastatic involvement within 16 months of presentation.

## Introduction

Melanoma of the skin had an incidence of roughly 23% of all new US cancer diagnoses in 2017 [1]. While the majority of melanomas are cutaneous lesions of the skin, only 4-5% of melanomas are extracutaneous [2]. One extracutaneous lesion, primary malignant melanoma (PMM) of the bladder, represents less than 0.2% of all melanomas [2-5], making this malignancy extremely rare. In regards to bladder tumors, 95% of all bladder tumors are typically urothelial carcinomas, with only 5% representing other rare primary cancers such as gastrointestinal stromal tumors, lymphoma, and neuroendocrine tumors [3]. Thus, PMM of the bladder represents both an exceptionally rare melanoma and an exceedingly rare bladder tumor.

Extracutaneous melanomas are extraordinarily rare, aggressive, and extremely lethal [2]. PMM of the bladder, a type of extracutaneous melanoma, typically presents with gross hematuria and/or other voiding symptoms such as dysuria or incontinence [2-5]. There is no apparent risk regarding gender at this time in regards to PMM of the bladder, and it affects both genders equally [3]. PMM typically presents in people over 50 years old [2], with a mean age of 60.6 years [3].

At this time, the PMM of the bladder has an unknown etiology and risk factors [4]. Both metastatic malignant melanoma and PMM of the bladder have similar histopathological features making their distinction and diagnosis difficult [3]. As of now, the diagnosis of PMM of the bladder is based on ruling out any other area for concerns of primary melanoma presence or prior regression of disease [3]. First-line treatment is surgery including radical or partial cystectomy and transurethral resection of bladder tumor (TURBT). Additional therapies including radiation, chemotherapy, and immunotherapy have been described [2-5]. Here, we report a patient who presented with gross hematuria, and after detailed workup, the diagnosis of PMM of the bladder was made.

## Case presentation

**Figure 1:**
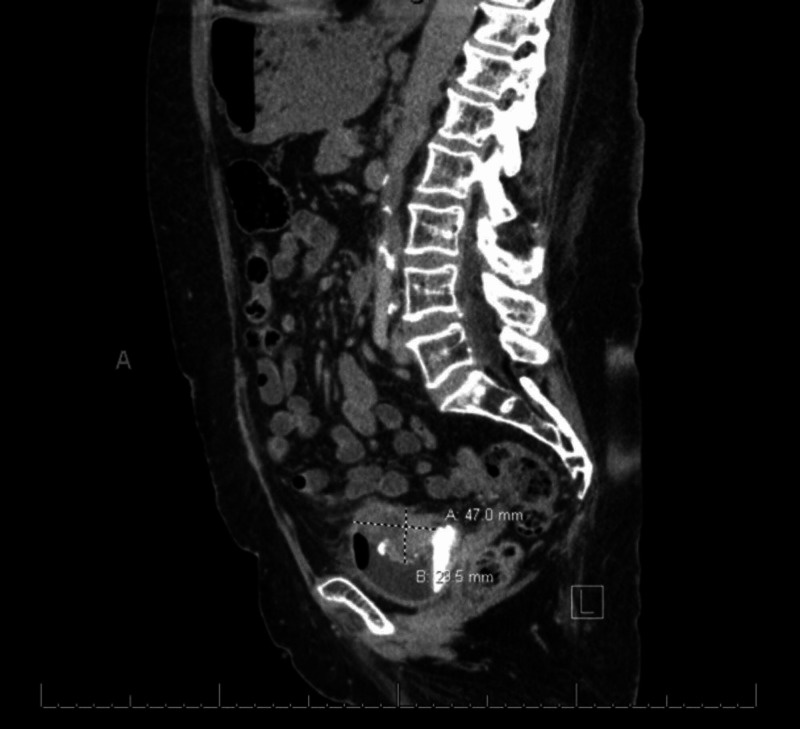
Initial CT urology during admission for gross hematuria, sagittal view demonstrating presence of calcified bladder mass. CT, computed tomography

**Figure 2:**
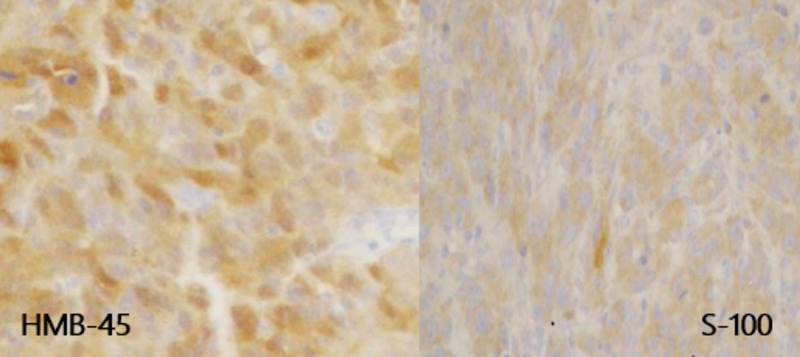
TURBT specimen stains positive for HMB-45 (left) and S-100 (right). TURBT, transurethral resection of bladder tumor

A 78-year-old female presented to the Emergency Department for new onset of incontinence and gross hematuria. Upon initial workup, computed tomography (CT) urogram (Figure 1) demonstrated a calcified mass in the bladder along with left hydroureteronephrosis. Shortly after the bladder mass diagnosis, the patient underwent a pelvic examination under anesthesia revealing negative findings, as well as a TURBT, and left ureteral stent placement. Pathological analysis of the resected tumor revealed PMM of the bladder as it demonstrated positive staining for HMB-45 and S-100 (Figure 2). Following the pathological diagnosis of the tumor resection, continued workup was done for melanoma. A fluorodeoxyglucose (FDG)-positron emission tomography (PET) was performed, which demonstrated positive uptake of FDG only in the bladder, and chest imaging was negative. The patient also had a suspicious lesion on the forearm of concern for possible melanoma; however, a biopsy of the forearm lesion showed dermatofibroma, thus ruling out melanoma. Therefore, PMM of the bladder was confirmed after extensive history, physical examination, and workup.

Eight months since her symptoms of hematuria and incontinence began, and approximately seven months following her initial diagnosis of PMM, the patient underwent radical cystectomy with ileal conduit and bilateral pelvic lymph node dissection, lysis adhesions, removal of the left ureteral stent, bilateral ureteral stent placement, hysterectomy and bilateral salpingo-oophorectomy, and excision of the anterior vaginal wall. The specimen was sent for analysis, and pathological analysis showed pT3N0M0 PMM of the bladder.

**Figure 3:**
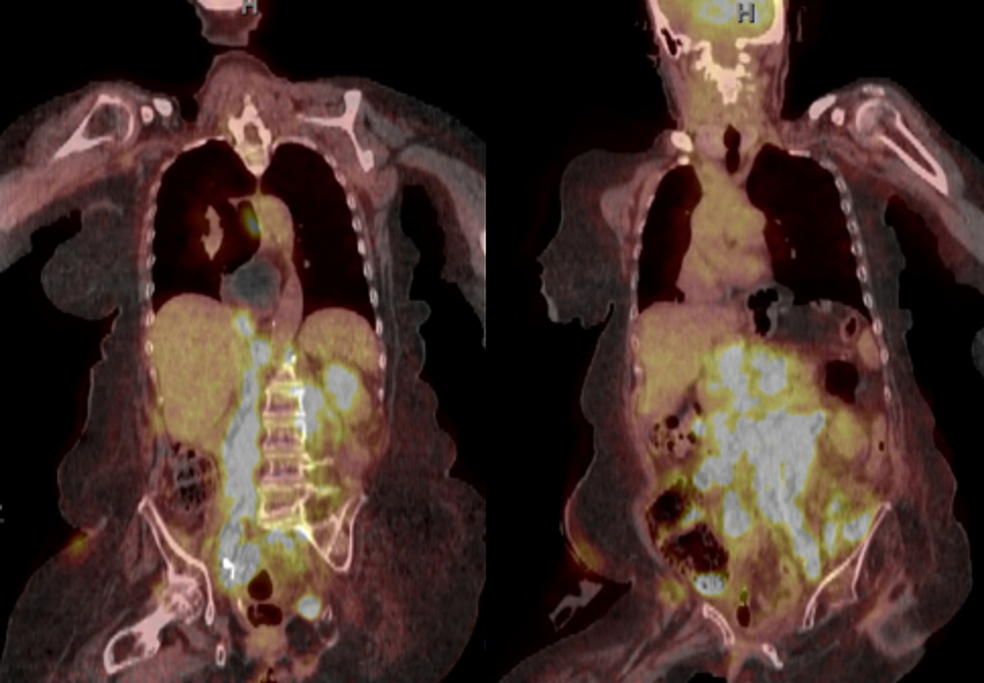
Coronal views, posterior to anterior from left to right, of the FDG-PET status post cystectomy, demonstrating diffuse metastasis at 16 months. FDG-PET, fluorodeoxyglucose-positron emission tomography

Six months later (14 months following the initial diagnosis), the patient presented with abdominal pain. A CT scan of the abdomen and pelvis showed new retroperitoneal lymphadenopathy and infrarenal occlusion of the aorta. A month later, FDG-PET CT (Figure 3) showed confluent bilateral adenopathy extending from the aortic hiatus into the pelvis, as well as positive lymph nodes in the chest. Pathological findings of the lymph node biopsy was positive for metastatic melanoma.

The patient had planned to proceed with adjuvant chemotherapy following cystectomy. However, the patient presented with recurrent abdominal pain and had a complicated hospital course. During that hospitalization the patient eventually succumbed to her metastatic disease.

## Discussion

Extracutaneous lesions of melanoma as discussed are very rare. Most cutaneous melanomas typically arise from genetic alterations of the melanocyte precursors due to direct ultraviolet (UV) radiation exposure leading to mutations and development of malignancy. However, these melanocyte precursors are also found in areas of squamous-covered mucous membranes such as the respiratory and urogenital tracts [4]. Mucosal melanomas have been described, but only account for 1.3-1.4% of all melanomas. Incidence of mucosal melanomas of the vagina represent 7.4%, penis 3.3%, urethra 1.8%, and bladder 0.2% of the cases [4]. As these particular squamous-covered mucous membranes are not exposed to UV radiation, the histopathogenesis and etiology of PMM of the urinary bladder remains unknown at this time [3,4].

To differentiate metastatic melanoma of the bladder from primary melanoma of the bladder, the following criteria have been used: (1) detailed history ruling out cutaneous, regressed, or visceral melanoma; and (2) recurrence pattern that is consistent with the primary origin of melanoma [2-5]. Histological and immunohistochemistry can help confirm the diagnosis of primary melanoma, but due to its highly variable morphological appearance, these methods themselves may not be definitive. A review of cases shows that positive expression of S-100, HMB45, and Melan-A can help identify bladder melanoma [4]. Further investigation and characterization of its morphological variability and cellular patterns (nested, spindle cell, small cell variants) is needed so that PMM of the bladder is not missed in its diagnosis and its histological variant subtypes are accurately described and subsequent therapies delineated.

PMM of the bladder has a poor prognosis because it presents at an advanced stage, has ambiguous pathological features making it difficult to diagnose, and is very aggressive, with two-thirds of patients succumbing to metastatic disease by three years [4,5]. In addition, there is a lack of data suggesting the best treatment options for this rare cancer. First-line therapy is surgery with cystectomy or TURBT [2], but for those with poor performance, evidence for alternatives is sparse. It appears that tumor stage and mitotic activity can aid in prognosis, and some authors suggest identifying if the malignancy contains a BRAF-activating mutation for therapeutic targeting may be of benefit [3,4]. Others have suggested immunotherapy after cystectomy with nivolumab as a successful treatment [2]. Overall, we have yet to define the best treatment regimen.

## Conclusions

Diagnosis and treatment of PMM of the bladder is difficult due to its similar histological presentation similar to that of metastatic melanoma, its advanced disease at presentation, aggressive nature and mortality, and rarity of cases with survival after treatment, as described above. At this time, there is no universally accepted method of treatment, but its poor prognosis is consistent throughout.

In conclusion, due to the rarity of PMM of the bladder, evidence regarding etiology, risk factors, histopathological diagnosis, and treatment options remains in question. Further analysis of all reported cases and head-to-head clinical trials regarding treatment options is warranted to adequately identify and describe these unknowns.
